# Transcriptomic analysis of stem cells from chorionic villi uncovers the impact of chromosomes 2, 6 and 22 in the clinical manifestations of Down syndrome

**DOI:** 10.1186/s13287-023-03503-4

**Published:** 2023-09-23

**Authors:** Salvatore Vaiasicca, Gianmarco Melone, David W. James, Marcos Quintela, Alessandra Preziuso, Richard H. Finnell, Robert Steven Conlan, Lewis W. Francis, Bruna Corradetti

**Affiliations:** 1 Department of Life and Environmental Sciences, Polytechnic University of Marche, Ancona, Italy; 2 Scientific Direction, IRCCS INRCA, Ancona, Italy; 3 Centre for NanoHealth, Swansea University Medical School, Singleton Park, Swansea, Wales, UK; 4 Department of Nanomedicine, Houston Methodist Research Institute, Houston, TX USA; 5 Center for Precision Environmental Health, Baylor College of Medicine, Houston, TX USA

**Keywords:** Chorionic villi, Mesenchymal stem cells, Down syndrome, Next-generation sequencing analysis

## Abstract

**Background:**

Down syndrome (DS) clinical multisystem condition is generally considered the result of a genetic imbalance generated by the extra copy of chromosome 21. Recent discoveries, however, demonstrate that the molecular mechanisms activated in DS compared to euploid individuals are more complex than previously thought. Here, we utilize mesenchymal stem cells from chorionic villi (CV) to uncover the role of comprehensive functional genomics-based understanding of DS complexity.

**Methods:**

Next-generation sequencing coupled with bioinformatic analysis was performed on CV obtained from women carrying fetuses with DS (DS-CV) to reveal specific genome-wide transcriptional changes compared to their euploid counterparts. Functional assays were carried out to confirm the biological processes identified as enriched in DS-CV compared to CV (i.e., cell cycle, proliferation features, immunosuppression and ROS production).

**Results:**

Genes located on chromosomes other than the canonical 21 (Ch. 2, 6 and 22) are responsible for the impairment of life-essential pathways, including cell cycle regulation, innate immune response and reaction to external stimuli were found to be differentially expressed in DS-CV. Experimental validation confirmed the key role of the biological pathways regulated by those genes in the etiology of such a multisystem condition.

**Conclusions:**

NGS dataset generated in this study highlights the compromised functionality in the proliferative rate and in the innate response of DS-associated clinical conditions and identifies DS-CV as suitable tools for the development of specifically tailored, personalized intervention modalities.

**Supplementary Information:**

The online version contains supplementary material available at 10.1186/s13287-023-03503-4.

## Background

Down syndrome (DS) is the most common chromosome-related disorder, with an incidence of 1 in 1000 live births worldwide according to the World Health Organization [1]. As a multisystem disease, DS is caused, in most cases, by meiotic non-disjunction of the maternal chromosome 21 and results in a third copy of the entire chromosome 21 in somatic cells [2–4]. The extra copy of the chromosome 21 gene set and the consequent gene dosage imbalance results in altered morphological development, leading to dysmorphic features (e.g., craniofacial abnormalities, hypotonia and cognitive impairment) [5] and often, associated pathological conditions. Common conditions include respiratory tract infections, autoimmune disorders, diabetes mellitus, celiac disease, thyroid impairment, gastrointestinal cardiac defects and, in some cases, leukemia [6].

While DS has been recognized as a clinical entity for about 150 years, effective therapies to improve speech and articulation, prevent cognitive decline, recover immune function and to prevent leukemia initiation remain to be developed for DS patients [7]. Only recently the in-depth molecular analyses have begun to reveal the utility of using molecular genetics and genomics as a promising exploratory approach to better understand DS pathogenesis [6, 8, 9]. Specifically, the current thrust of research on DS is based on the premise that understanding mechanisms at the genetic and molecular level will provide a rational basis for the development of effective therapeutic and preventive interventions [10]. The transcriptome of DS-derived cells offers a more comprehensive overview of the molecular traits associated to DS and could assist in the identification of candidate genes (within or outside of chromosome 21) to be potentially considered as therapeutic targets [11].

To date, molecular studies have been primarily focused on the gain of function genes secondary to the extra chromosome 21 and their roles in determining the specific DS phenotype, compared to an euploid individuals [12, 13]. Several studies identified differentially expressed genes (DEGs) spread over all of the chromosomes, not limited to chromosome 21, which contribute to the DS phenotype [8, 14, 15]. Among them, a study identified complex intrachromosomal functional gene re-adjustments with 65 up-regulated and 111 down-regulated genes in chromosome 21 [16], which include those that are related to central nervous system development (8 genes) and extracellular matrix organization (11 genes), influencing the normal development and communication among neuronal cells. Another study has shown that the trisomy not only determines an imbalance in gene expression associated to chromosome 21, but it also induced differences in gene transcripts situated on other chromosomes [13]. The transcriptomic analysis in monozygotic twins discordant for trisomy 21 revealed significant gene expression changes in other chromosome (namely 1, 6, 11, 16, 17 and 19), indicating that the molecular mechanisms activated in DS compared to euploid individuals is more complex than previously thought [16]. DS studies in human patients are limited for obvious ethical reasons, resulting in the use of differential models that have been proposed to study this syndrome. Mouse models or induced pluripotent stem cells (iPSCs) cells are a few examples of the strategies developed so far to provide new insights into the DS phenotype and represent suitable tools for the identification of potential targets for therapeutic options. On the other hand, modified iPSCs present therapeutic limitations related to the viral vector integration into the host genome and causing genomic and epigenetic changes [17]. Mesenchymal stem cells (MSCs) from extraembryonic tissues are easily collected before, during and after the birth, without requiring invasive procedures or experiencing ethical limitation in comparison with those associated with their adult and embryonic counterparts [18]. In this study, we propose MSCs isolated from a gestational tissue, the chorionic villi (CV), sampled during prenatal diagnosis, for a personalized and comprehensive functional genomics-based understanding of this complex clinical condition which will provide new knowledge-based insights for future clinical treatment of DS. We used next-generation sequencing (NGS) analysis to compare the whole transcriptomic profiles of MSC populations isolated from CV derived from women carrying fetuses diagnosed with DS (DS-CV) with whole transcriptomes of their euploid counterparts (CV). Experimental validation of the biological processes identified as enriched in DS-CV compared to CV included the assessment of crucial pathways related to the typical MSC phenotype (i.e., cell cycle and proliferation features) as well as those responsible for the marked reactivity to external stimuli (e.g., production of reactive oxygen species, ROS) and enhanced immunosuppressive capability attributed to DS-CV.

## Methods

### Establishment of cell cultures

Chorionic villi (CV) samples were obtained from pregnant women undergoing prenatal diagnosis between the 10th and the 13th week of gestation at the Cytogenetic Laboratory Children's Hospital Salesi (Ancona, Italy) upon informed written consent for the use of tissue for research purposes. The study was approved by the Regional Institutional Review Board (Comitato Etico Regione Marche) and was conducted in accordance with the Declaration of Helsinki. CV were carefully separated from maternal decidua using sterile fine forceps to avoid the contamination and washed with 1 × phosphate-buffered saline (PBS) to remove any blood clots and subjected to mechanical treatment to obtain small fragments (3 mm^2^). Finally, the sample was transferred into flasks where the cells were allowed to adhere and migrate out of the tissue. Primary MSCs isolated from healthy women (CV) and women carrying fetuses with diagnosed DS (DS-CV) were expanded in standard culture medium consisting of high-glucose Dulbecco's modified eagle medium (HG-DMEM, Corning) supplemented with 10% Fetal Bovine Serum (FBS), 1% L-glutamine and 2% antimitotic-antibiotics (A/A, EuroClone S.p.A). Human monocytic cells (THP-1 cells) used in this study were purchased from ATCC. THP-1 cell culture was established in Roswell Park Memorial Institute-1640 (RPMI-1640) media (Gibco) supplemented with 10% FBS (Corning), 1% L-glutamine and 2% A/A. Cell cultures were maintained at 5% CO_2_ atmosphere and 37^◦^C temperature.

### RNA sequence analysis performed using next-generation sequencing (NGS)

#### RNA extraction and quality check

Total RNA was extracted from CV- and DS-CV at passage 2 (P2) using total RNA Purification Plus Kit (Norgen) and processed by the Functional Genomic Center (University of Verona). RNA concentration and integrity were assessed using the RNA 6000 Nano Kit (Agilent Technologies). RNA samples that showed an integrity number (RIN) > 9 were used for the NGS.

#### RNA-Seq library preparation

RNA-Seq library preparation was performed using the TruSeq stranded mRNA kit (Illumina) from 1 μg of RNA per sample. Library size was assessed by capillary electrophoretic analysis with the Agilent 4200 Tape station. RNA libraries were analyzed on an Illumina NextSeq 500 sequencer using 75nt single reads. The quality of the reads was checked using the software FastQC (http://www.bioinformatics.babraham.ac.uk/projects/fastqc/)**,** discarding those reporting more than 50 bp with low scores. Subsequently, through the software Scythe (https://github.com/vsbuffalo/scythe), adaptor contamination was removed, and the reads showing low quality were modified at their endings using the software Sickle (https://github.com/vsbuffalo/sickle). Filtered reads were aligned to the Human reference genome GRCh38 using HISAT2 [19]. Differential analysis was performed using the R bioconductor package DESeq2 [20], contrasting the 3 CV samples with the 3 ds-CV samples. Raw and processed data are deposited in the Gene Expression Omnibus (GEO) database with accession number GSE184450.

#### Heatmap and volcano plot

To visualize the differentially expressed genes (DEGs) between CV and DS-CV, heatmap and volcano plot were created using ggplot2 package [21]. Chord diagrams were generated using the GOPlot R package [22]. The criteria of DEGs were adjusted *p*-value (Padj) < 0.05. In the heatmap and in the volcano plot, the DEGs are displayed as color-coded; red represents over-expression while blue under-expression. In the heatmap the Euclidean distance of samples was used, while in the volcano plot mean of normalized counts was used.

#### Pathway and gene ontology

Pathway analysis on all significantly dysregulated genes was performed using the WebGestaltR [23] package, with gene set enrichment analysis (GSEA) [24] enrichment method. The whole genome was used as the background, with gene sets REACTOME pathway [25], KEGG pathway [26], GO biological processes [27] and CytogenicBand analyzed for enrichment.

#### Protein–protein interactions

Protein–protein interactions were obtained using the Cytoscape StringApp [28], which uses STRING-DB [29] for protein–protein interaction data. For each pair of chromosomes considered, the significantly dysregulated genes were imported into the StringApp, which automatically generated protein networks based on interactions between proteins annotated in STRING-DB. The largest cluster of interacting proteins was selected, and enrichment analysis was performed within the StringApp, for Gene Ontology Biological Process [27], Gene Ontology Cellular Component [27] and REACTOME pathway sets [30], with the whole genome used as the background.

### Characterization of MSCs from CV and DS-CV

#### Morphology

Morphology was evaluated through fluorescence microscopy (OLYMPUS BX51, equipped with the Spot Advanced software). ActinGreen (Life Technologies) and Hoechst (Sigma-Aldrich) were used to highlight the cytoskeleton and the nucleus, respectively. Briefly, cells were seeded at 1.4 × 10^4^/well in 8 chamber-slides (Corning® BioCoat™ CultureSlides) and let adhere. Cells were then washed twice in PBS and immediately fixed with 4% paraformaldehyde (PFA) in PBS for 15 min. Subsequently, cells were washed for three times in PBS and permeabilized with 0.1% Triton X-100 (Sigma-Aldrich) (in PBS) for 10–15 min. After a second round of washing, MSCs were blocked in 1% bovine serum albumin (BSA)/PBS for 30 min at room temperature. Cells were then incubated with ActinGreen for 20 min at room temperature in the dark. Five minutes before visualization, cells were incubated with Hoechst (1 μg/ml).

#### Expression of MSC-associated markers by flow cytometry

Cell phenotype was analyzed according to the published criteria [31]. A total of 5 × 10^5^ cells (at P2) were collected, fixed with 75% ethanol and incubated for 20 min with 0.5% BSA/PBS to block non-specific sites. Samples were then centrifuged at 500 × g for 5 min at 20 °C and stained with fluorescently labeled antibodies. Antibodies include phycoerythrin (PE)-conjugated anti-ecto-5′-nucleotidase (PE-CD73; BioLegend), fluorescein isothiocyanate (FITC)-conjugated anti-thymocyte differentiation antigen 1 (FITC-CD90; BioLegend) and fluorescein isothiocyanate (FITC)-conjugated anti-glycoprotein CD44 (FITC-BioLegend), and allophycocyanin (APC)-conjugated anti-integrin β1 (APC-CD29; BioLegend). Cells were incubated for 45 min at room temperature in the dark, then washed twice with filtered PBS to remove the excess of antibody and analyzed using Guava Easycyte Millipore flow cytometer with GUAVASOFT 2.2.3 software.

#### Differentiative potential

To test their multipotent differentiative potential, CV and DS-CV at P3 were seeded at a density of 1 × 10^3^/cm^2^ in six-well tissue culture dishes. For osteogenesis, cells were cultured in HG-DMEM, supplemented with 10% FBS, 100 U/ml penicillin, 100 μg/ml streptomycin, 0.25 μg/ml amphotericin B, 2 mM/l L-glutamine, 10 mM β-glycerophosphate (Sigma, 50020), 0.1 μM dexamethasone (Sigma, D2915) and 250 μM ascorbic acid (Sigma, A8960). For adipogenic differentiation, cells were cultured in HG-DMEM, supplemented with 10% FBS, 100 U/ml penicillin, 100 μg/ml streptomycin, 0.25 μg/ml amphotericin B, 2 mM/l L-glutamine, 10 μg/ml insulin (Sigma I-6634), 150 μM indomethacin (Sigma I-7378), 1 μM dexamethasone and 500 μM IBMX (3-isobutyl-methyl-xanthine, Sigma I-7018). Induced cells were incubated for two weeks at 38.5 °C with 5% CO_2_. Non-induced control cells were cultured for the same time with standard medium (HG-DMEM supplemented with 10% FBS, 100 U/ml penicillin, 100 μg/ml streptomycin, 0.25 μg/ml amphotericin B, 2 mM/l L-glutamine). The presence of calcium deposits in differentiated cells was verified after 7 and 14 days of induction by von Kossa staining, whereas Oil Red-O staining (Sigma, O0625) was used to identify lipid droplets in the cytoplasm. Expression of specific genes was performed as reported in section “Gene expression analysis” to confirm occurred differentiation.

#### Clonogenicity

CV and DS-CV-derived MSCs (at P0) were seeded at different densities (namely, 3.5 × 10^2^, 1 × 10^3^, 3.5 × 10^3^ cells/cm^2^) in six-well plates for 2 weeks in stem cell standard culture medium. At the end of the 2 weeks culture period, colonies were fixed with 1% paraformaldehyde (PFA), stained with Giemsa at room temperature (RT) and washed twice. Colonies formed by 15–20 nucleated were counted with an inverted microscope (Meiji Techno).

### Proliferation of MSCs from CV and DS-CV

*Growth curve* were produced by seeding CV- and DS-CV (at P2) at the density 9.5 × 103 cells/well into six-well tissue culture polystyrene dishes (EuroClone). Every 2 days, over a 15-day culture period, cells were trypsinized and counted as previously reported [32]. Analysis was performed in triplicate.

### Cell cycle analysis

To determine possible differences in cell cycle between MSCs from CV- and DS-CV, the distribution of cells in the major phases of the cycle (subG0, G1, S, G2/M) was examined using flow cytometry. One million cells (per group) were harvested (at P2) and were fixed by overnight incubation with cold 70% ethanol. Cells were subsequently resuspended with a staining solution containing propidium iodide (PI, 40 μg/ml) and RNase (100 μg/ml) in PBS. At least 20,000 events/sample were acquired through a Guava Millipore cytometer. The percentage of cells in each phase was established using FlowJo software, through a red channel.

### Assessment of CV and DS-CV immunosuppressive potential

To assess the immunosuppressive potential of CV- and DS-CV, cells (at P2) were seeded at the density of 2 × 10^4^ in 24-well plates and cultured for 24 h at 37 °C before stimulation with pro-inflammatory cytokines. Soluble recombinant human tumor necrosis factor-alpha (*TNF-α)* and interferon gamma (*IFN-γ)* (Peprotech) were used to simulate an inflammatory environment at the concentration of 20 ng/ml. After 24- and 48-h cells were harvested and processed for molecular analysis as reported in Gene expression analysis paragraph.

### Reactive oxygen species (ROS) detection on MSCs from CV and DS-CV

Cellular oxidative stress was evaluated through the reactive 2′,7′-dichlorodihydrofluorecein (H2-DCFDA, Sigma-Aldrich) assay. CV- and DS-CV were trypsinized, washed two times with cold PBS, suspended with PBS containing the probe at the working concentration of 10 μM and incubated for 30 min at 37°. Cells were subsequently washed twice in 1 × PBS and stained with 10 μg/ml PI to exclude dead cells from the analysis. The mean fluorescence intensity of cells emitting in the green (H2-DCFDA) and red (PI) channel was analyzed using Guava Easycyte Millipore flow cytometer with GUAVASOFT 2.2.3 software.

### Assessment of CV and DS-CV immunomodulatory potential

CV and DS-CV MSCs (at P2) were seeded at the density of 3 × 10^3^ cells/cm^2^ in T75 flasks and cultured to 70–80% confluence. Cells were then washed thoroughly 3 times with Hank's balanced salt solution (HBSS) (without Ca2^+^ and Mg2^+^) (Gibco) and replenished with standard culture medium supplemented with 10% exosome depleted FBS (Gibco). Cells were maintained in standard conditions (5% CO_2_; 37 °C) for 24 and 48 h. The media conditioned by CV and DS-CV MSCs was then collected, centrifuged at 500 × g for 10 min at 4 °C to remove cell debris and frozen in aliquots at − 80 °C until further analyses. The role CV and DS-CV MSCs play in modulating the behavior/phenotype of immune cells was assessed on monocytic cells THP-1. THP-1 cells were plated at density 5 × 10^5^ in 24-well plates and exposed for 24 and 48 h to the conditioned media produced by CV and DS-CV. At each time point cells were harvested and processed for molecular analysis, as reported in the gene expression analysis paragraph.

### Gene expression analysis

Gene expression evaluation was performed using the human specific oligonucleotide primers described in Table 1. Primers were designed using open-source Primer-BLAST (National Center for Biotechnology Information, NCBI) across an exon–exon junction to avoid genomic DNA amplification. Manual corrections were made to improve specificity. Total RNA was isolated using TRI-reagent (Sigma) and purified from genomic DNA through digestion with DNAse (Sigma). RNA concentration and purity were measured using a NanoDrop ND1000 spectrophotometer (NanoDrop Technologies). The cDNA was synthesized from 500 ng of total RNA using the PrimeScript RT Master Mix (TAKARA) and qPCR was run in the Step One Plus Real-time PCR thermocycler (Applied Biosystems) using the commercially available PowerUp SYBER Green Master Mix (Applied Biosystems).

**Table 1:** Oligonucleotide sequence, melting temperature (*T*_m_), transcript length (base pairs, bp) of the primers used to evaluate the expression of MSC-, differentiation- and inflammation-associated genes

Genes	Sequences (5′ → 3′)	*T*_m_ (°C)	Product size (bp)
*Mesenchymal*			
CD44 molecule (*Cd44*)	S: GGAGCAGCACTTCAGGAGGTTAC A: GGAATGTGTCTTGGTCTCTGGTAGC	63	129
5’-nucleotidase, ecto (*Cd73*)	S: GCTCTTCACCAAGGTTCAGC A: GTGGCTCGATCAGTCCTTCC	59	203
Thy-1 cell surface antigen (*Cd90*)	S: CTTTGGCACTGTGGGGGTGC A: GATGCCCTCACACTTGACCAG	61	211
Endoglin (*Cd105*)	S: CCTGGAGTTCCCAACGGGCC A: GGCTCTTGGAAGGTGACCAGG	62	186
Integrin beta-1 (*Cd29*)	S: CGTGGTTGCCGGAATTGTTC A: AGTTGTCACGGCACTCTTGT	60	162
*Pro- and anti-inflammatory*			
Tumor necrosis factor-alpha (*Tnf-α*)	S: TCTGGCCCAGGCAGTCAGATC A: TACAGGCCCTCTGATGGCACC	64	180
Interleukin 6 (*Il-6*)	S: AACTCCTTCTCCACAAGCGC A: ATGCCGTCGAGGATGTACCG	60	188
Interleukin 1-beta (*Il-1β)*	S: TGCTCTGGGATTCTCTTCAGC A: CTGGAAGGAGCACTTCATCTG	60	164
Prostaglandin E-receptor 2 (*Pge-2)*	S: GGAAGGAGAAAGCTCGCAAC A: TGAGCCAGTACTTATTGCCG	58	173
Transforming growth factor beta (*Tgf-β)*	S: TGGTCATGAGCTTCGTCAAC A: TCTCATTGTCGAAGCGTTCC	58	171
Cyclooxygenase 2 (*Cox-2*)	S: TGAGTTATGTGTTGACATCCAG A: TCATTTGAATCAGGAAGCTGC	62	190
*Differentiation (osteogenesis and adipogenesis)*			
RUNX family transcription factor 2 (*Runx2*)	S: GGTTAATCTCCGCAGGTCACT A: CACTGTGCTGAAGAGGCTGTT	60	143
Bone gamma-carboxyglutamate protein* (Bglap)*	S: TCACACTCCTCGCCCTATTG A: TCGCTGCCCTCCTGCTTG	61	137
Adiponectin* (Adipoq)*	S: CCCAAAGAGGAGAGAGGAAGCT A: GCCAGAGCAATGAGATGCAA	60	73
Peroxisome proliferator-activated receptor γ *(PPAR-γ)*	S: ATTGACCCAGAAAGCGATTC A: CAAAGGAGTGGGAGTGGTCT	58	154
*Housekeeping*			
Glyceraldehyde-3-phosphatase dehydrogenase (*Gapdh*)	S: TCCACTGGCGTCTTCACC A: GGCAGAGATGATGACCCTTT	68	78

S, Sense primer; A, Antisense primer; *T*_m_, Melting temperature; bp, Base pairs

### Statistical analysis

Statistical analysis was performed using GraphPad Instat 3.00 for Windows (GraphPad Software). Three replicates for each experiment (growth curve, colony forming unit, cell cycle, differentiative potential and flow cytometric analysis) were performed. Results are reported as mean ± standard deviation (SD). One-way analysis of variance for multiple comparisons by the Student–Newman–Keuls multiple comparison test was used to assess differences between groups. Differences were considered statistically significant for *p* values < 0.05. For quantitative PCR data, nonparametric tests were used.

## Results

### Transcriptome analysis reveals DEGs between CV and CV-DS

To investigate transcriptomic differences between CV and DS-CV, we conducted a whole transcriptome NGS analysis (RNA-Seq) on three biological replicates for each MSC population. Quality control analyses were performed on raw Illumina reads using FastQC to confirm the quality of the sequencing data (Table 2). Large percentages of uniquely mapped reads that align to exactly one location within the reference genome (> 98.9%) confirmed the high quality of the library preparation [33]. Following current RNA-Seq guidelines, these parameters were deemed acceptable for pursuing further analysis of RNA-Seq data obtained from all samples (Additional file 1).

**Table 2:** Gene reads obtained during the NGS analysis of chorionic villi isolated from women carrying healthy (CV) and Down syndrome fetuses (DS-CV)

Name	Total reads	Mapped reads (%)	Unique mapping (%)	Multimapping reads (%)
CV_1	32,119,080	99.04	91.24	7.81
CV_2	29,440,066	99.02	90.16	8.86
CV_3	33,087,547	99.03	90.76	8.27
DS-CV_1	34,198,925	99.05	91.57	7.48
DS-CV_2	35,575,785	98.96	91.14	7.82
DS-CV_3	37,819,995	98.99	91.65	7.34

Raw and aligned reads indicate the reads before and after the bioinformatics processes, respectively. Three biological replicates were considered in the analysis

**Fig. 1:**
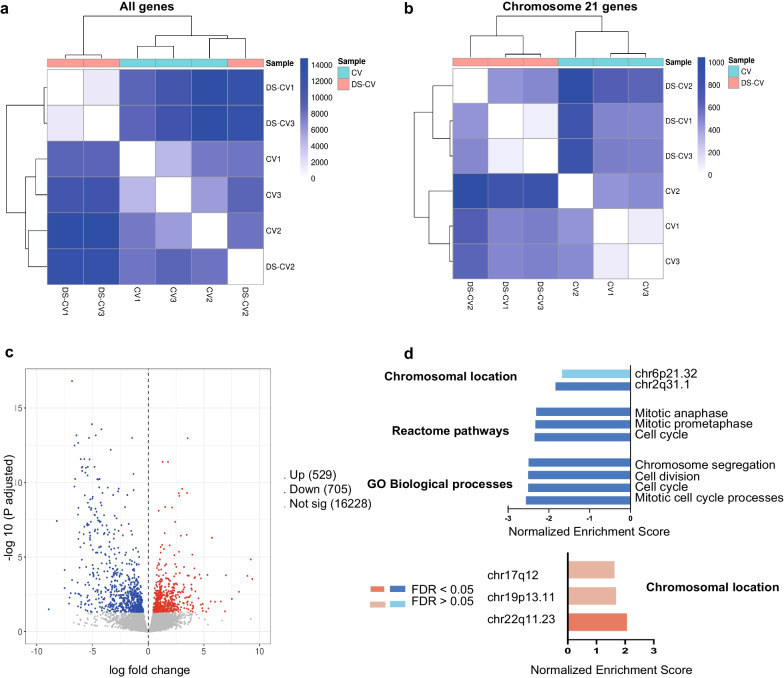
Transcriptome analysis reveals DEG between CV and CV-DS. **a** Heatmap of Euclidean distance scores of normalized genes counts on all chromosome for DS-CV versus CV. Dendrograms show hierarchical clustering results. **b** Heatmap of Euclidean distance scores of normalized gene counts on chromosome 21 for DS-CV versus CV. **c** Volcano plot displaying the log fold change and *p*-value of genes in DS-CV samples against CV samples. Red markers indicate significantly up-regulated genes and blue markers indicate significantly down-regulated genes (Padj < 0.05). Gray markers indicate genes where Padj > 0.05. **d** Gene set enrichment analysis (GSEA) for GO biological process and REACTOME pathways for all DEGs and chromosomal locations for DEGs for DS-CV versus CV

Plotting the Euclidean distances revealed fundamental differences and similarities between CV and DS-CV biological samples (Fig. 1a). All three CV biological replicates were found within optimal proximity to one another and significantly separated from their DS-CV counterparts. On the contrary, only two of the DS-CV samples grouped together, with the third replicate isolated from CV and DS-CV samples. To further investigate this phenomenon, we performed a hierarchical clustering heatmap including only genes expressed on chromosome 21. In this case, all three DS-CV samples clustered together (Fig. 1b). DS-CV samples on chromosome 2, 6 and 22 did not clustered, as shown in the correlation plots reported in Additional file 2: Fig. S1.

Differential analysis between CV and DS-CV samples identified a total of 1238 significantly altered transcripts (adjusted *p*-value [padj] < 0.05; |log2foldchange|> 1) (Fig. 1c, Additional file 3: Table S1). Specifically, 529 DEGs were up-regulated in DS-CV samples compared with CV, whereas 705 DEGs were down-regulated (Fig. 1c and Additional file 3: Table S1). Interestingly, only 30 up-regulated and 6 down-regulated transcripts originated from genes located in chromosome 21 (5.7% and 0.9% of the total DEGs, respectively). To gain further insight into which pathways were significantly enriched in DS-CV samples, we performed extensive in silico GSEA [24]. GSEA highlighted a significant down-regulation of processes related to cell cycle when testing the enrichment of GO biological processes [29, 34] and reactome pathways [35] (FDR < 0.05) (Fig. 1d and Additional file 4: Fig. S2). GSEA also pinpointed the presence of enriched cytobands (FDR < 0.1), including a down-regulated chromosome region 2q31.1 (Additional file 5: Table S2) and another such chromosome region 6p21.32 32 (Additional file 6: Table S3), along with an up-regulated chromosome region 22q11 (Additional file 7: Table S4) (Fig. 1d). The chromosome 2q31.1 band harbored a significant number of cell cycle-related genes including *Cdca7* (Fold change [FC] = -3.9), *Hoxd8* (FC = -5.4) and *Klhl23* (FC = -4.2). In addition, the metabolism-related glutathione catabolic process (FDR = 3.8e−4) and cofactor metabolic process (FDR = 0.003) genes were enriched. On cytoband chromosome 6p21.32, we identified three significantly down-regulated members of the major histocompatibility complex (MHC-II) genes, *Hla-dra* (FC = -4.8), *Hla-dpa1* (FC = 6.0) and *Hla-dpb1* (FC = 6.3) in DS-CV samples. In addition, we also explored protein–protein interactions between significantly dysregulated genes on chromosome 21 and the chromosomes containing significantly enriched cytobands (2, 6 and 22) in our DS-CV samples, generated networks of genes that were dysregulated within our DS-CV samples. A significant portion of the detected DEGs were primarily linked to chromosomes 2, 22 and 6 (Fig. 1d and Additional file 8: Fig. S3). Chromosomes 2 and 22 including all their gene clusters involved in cell proliferation and cell cycle regulation showed a significantly altered activity that merited further investigation. Similar analysis has also been conducted for the gene clusters related to the immune response on chromosome 6 (Additional file 5: Table S2, Additional file 6: Table S3, Additional file 7: Table S4).

### DEGs located on chromosome 2 and chromosome 22 influence the DS-CV proliferative properties

**Fig. 2:**
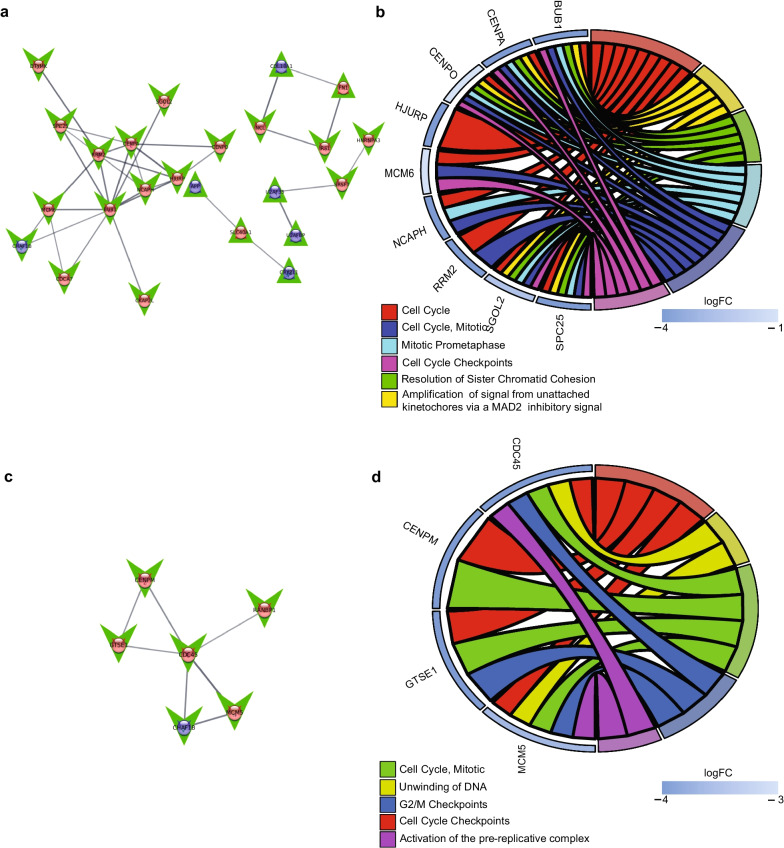
DEGs located on Chr 2 and Chr 22 influence the DS-CV proliferative properties. **a** Network diagram showing interactions between protein products for genes found to be significantly dysregulated on chromosome 21 and chromosome 2 (string score > 0.7). Blue nodes indicate genes on chromosome 21, red nodes indicate genes on chromosome 2, green upward pointing triangles indicate up-regulated genes, and green downward pointing arrows indicate down-regulated DEGs. Edge thickness indicates protein–protein interaction strength based on string score [0, 1]. **b** Chord diagram showing enriched pathways for genes in chromosome 21/chromosome 2 networks, showing DEGs involved in each pathway. Color of outer bars on genes indicates log fold change (note all genes in pathways are located on chromosome 2). **c** Network diagram showing interactions between protein products for genes found to be significantly dysregulated on chromosome 21 and chromosome 22 (string score > 0.7). Blue nodes indicate genes on chromosome 21, red nodes indicate genes on chromosome 22, green upward pointing triangles indicate up-regulated genes, and green downward pointing arrows indicate down-regulated DEGs. Edge thickness indicate protein–protein interaction strength based on string score [0, 1]. **d** Chord diagram showing enriched pathways for genes in chromosome 21/chromosome 22 network, showing DEGs involved in each pathway. Color of outer bars on genes indicates log fold change (note all genes in pathways are located on chromosome 22)

Figure 2a depicts a network diagram of the largest networks of interacting proteins dysregulated on chromosome 2 and chromosome 21 in DS-CV samples. These networks contain a total of 24 nodes and 33 edges, with 4 nodes up-regulated and 1 down-regulated on chromosome 21 and 2 up-regulated and 17 down-regulated on chromosome 2. The annotations of protein–protein interactions performed by the STRING cytoscape platform identified pathways involving these genes [35]. The five enriched pathways with the lowest FDR and corresponding genes in the network are shown in Fig. 2b. Genes identified in these pathways are located only on chromosome 22. Highlighted pathways were predominantly related to cell cycle, cell cycle checkpoints and key processes involved in the cell cycle, indicating that its function is altered in DS-CV cells when compared with CV cells. The largest network of interacting dysregulated genes on chromosome 21 and 22 is shown in Fig. 2c. The network contains a total of 6 nodes and 7 edges, with all nodes down-regulated on both chromosomes 21 and 22. The enriched pathways and corresponding genes are shown in Fig. 2d.

### Functional studies show striking proliferative differences between CV and DS-CV

**Fig. 3:**
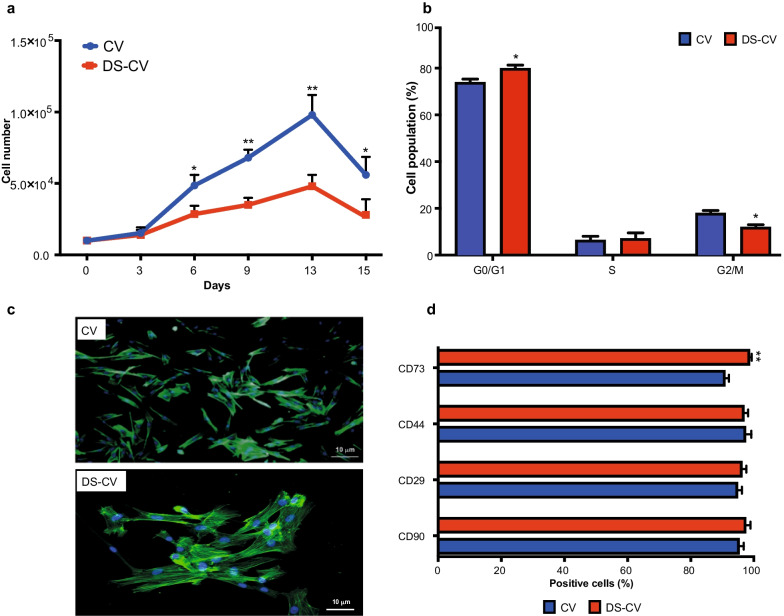
Evaluation of the CV- and DS-CV features. **a** Growth curve produced for CV (blue line) and DS-CV (red line) at P2 over a 15-day period. Results are reported as average of three biological replicates ± standard deviation (* = *p* < 0.05, ** = *p* < 0.01). **b** Cell cycle analysis performed on CV and DS-CV. Data obtained are the comparison of CV and DS-CV cells. Data are reported as the average of the percentage of cells distributed in the G0/G1, S and G2/M phase ± SD (*n* = 3). Results are reported as an average of three biological replicates ± standard deviation. **c** Cell morphology visualized through identification of phalloidin (green) in CV (top) and DS-CV (bottom). Cell nucleus is stained using DAPI (blue). Images taken at 10 × magnification, scale bar: 10 µm. **d** Percentage of cells positive for the MSC-associated markers studied (CD73, CD44, CD29 and CD90) in CV (blue) and DS-CV (red). Results are presented as percentage of marker-positive cells and as average of three independent experiments ± standard deviation (** = *p* < 0.01)

As shown in Fig. 3, significant differences (*p* < 0.05 and *p* < 0.01) were found between CV and DS-CV in their proliferative properties. The growth curve shows a similar lag phase (until day 3) between the two lines analyzed. Following the lag phase (days 4–13), we noticed a significant discrepancy in the growth rate of CV compared to DS-CV. At day 6 the number of cells produced by CV was almost twice that observed in DS-CV (4.8 × 10^4^ ± 7.5 × 10^3^ vs 2.8 × 10^4^ ± 2.0 × 10^3^, respectively). This trend was maintained until the end of the log phase when the cell growth plateaued. At the latest time point (day 15), a significant difference (*p* < 0.05) was still evident between the two groups, with 5.6 × 10^4^ ± 1.2 × 10^4^ and 2.8 × 10^4^ ± 1.1 × 10^4^ for CV and DS-CV, respectively (Fig. 3a). Cell cycle analysis reveals important growth characteristics in eukaryotic cells. Our data indicated a statistically significant increase in the percentage of cells in G0/G1 (+ 5%) and G2/M (+ 6%) phase in DS-CV compared to CV (Fig. 3b). No differences were observed for the S phase S (+ 1.6% in DS-CV compared to CV). Furthermore, while CV and DS-CV display a fibroblast-like morphology, slight shape differences became noticeable: DS-CV MSCs were more elongated compared to their euploid counterparts (Fig. 3c). Flow cytometry revealed that in both cell line preparations, over 90% of cells were positive for MSC-associated markers (CD90, CD29, CD44 and CD73) at protein level, a significant increase (*p* < 0.01) in the percentage of CD73 + DS-CV compared to CV (98.8 ± 0.5 vs 91 ± 1.1, respectively) (Fig. 3d).

### CV and CV-DS show clonogenic and differentiative potential

Before proceeding with subsequent analyses, CV and CV-DS were also tested for clonogenicity and plasticity. The number of cell colonies formed was counted at P0 after seeding cells at different density/cm^2^. DS-CV display a clonogenic capacity similar to their euploid counterparts, with a direct correlation between the increase of CFU frequency and the increase of cell seeding density (Table 3).

**Table 3:** Colony forming unit (CFU)—fibroblastic like capability of MSCs isolated from CV and DS-CV at different seeding density (cells/cm^2^)

Density cells/cm^2^	Total cells	CFU	1 CFU each
*CV*			
350	3325	0	0
1000	9500	3 ± 0.3	3166
3500	32,860	15 ± 0.7	2190
*DS-CV*			
350	2175	0	0
1000	7230	2 ± 0.2	3626
3500	31,487	8 ± 0.6	3930

Data are reported as the average of three biological replicates ± standard deviation

Multipotent differentiation was evaluated toward the osteogenic and adipogenic lineages (Additional file 9: Fig. S4). Over two weeks of culture in osteogenic induction medium, CV and DS-CV distinctly changed their morphology and were surrounded by calcium deposits positive to von Kossa staining (Additional file 9: Fig. S4a). Expression of osteogenesis associated genes confirmed the occurred differentiation in both cell types with euploid MSCs showing a more marked specification process. Expression of the transcription factor *Runx-2* was assessed around 7.08 ± 1.23 and 4.22 ± 0.32 for CV and DS-CV, while the expression of the osteogenic marker *Bglap* showed values that assessed around 9.93 ± 1.32 and 3.13 ± 0.98, respectively. In controls, the same changes were not observed. A similar trend was observed with regard to adipogenesis. Both cell lines were found to differentiate to adipocytes (Additional file 9: Fig. S4b), as shown by the presence of lipid vacuoles. Gene expression confirmed occurred differentiation and significant extents between the two cell lines. Expression of the transcription factor *PPAR-γ* was found to be significantly increased compared to control, with values assessed at 257.53 ± 8.76 and 79.32 ± 9.21 for CV and DS-CV, respectively, while the expression of the adipogenesis associated marker adiponectin was found to be 11.54 ± 2.47 and 7.40 ± 2.12 for the same samples.

### DEGs located on chromosome 6 confer a marked potential to react to external stimuli

**Fig. 4:**
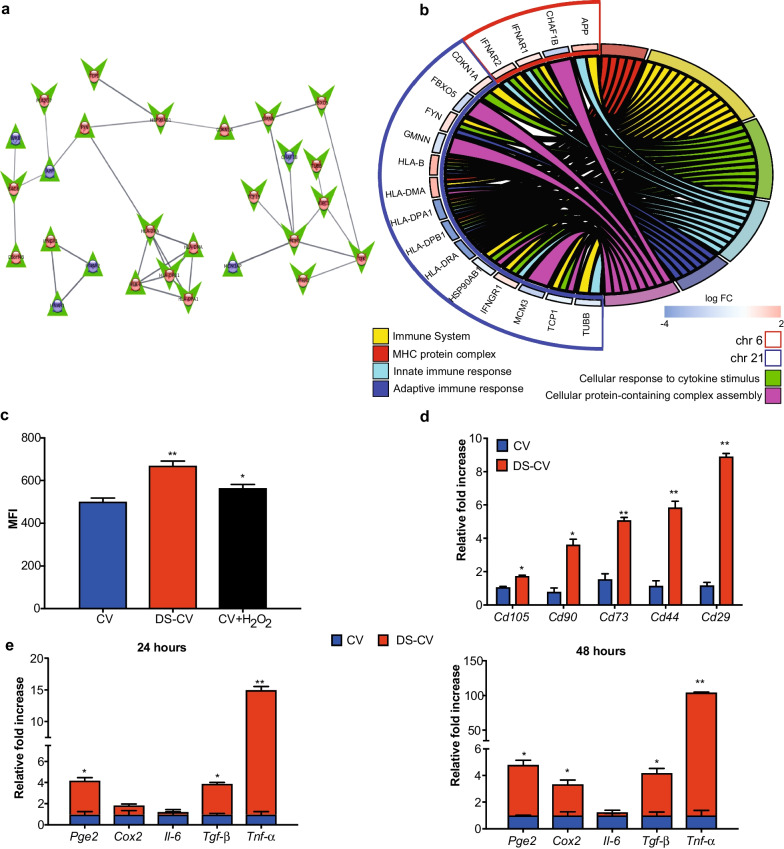
DEGs located on Chr 6 confer a marked potential to react to external stimuli. (**a**) Network diagram showing interactions between protein products for genes found to be significantly dysregulated on chromosome 21 and chromosome 6 (string score > 0.7). Blue nodes indicate genes on chromosome 21, red nodes indicate genes on chromosome 6, green upward pointing triangles indicate up-regulated genes, and green downward pointing arrows indicate down-regulated DEGs. Edge thickness indicates protein–protein interaction strength based on string score [0, 1]. **b** Chord diagram showing enriched pathways for genes in chromosome 21/chromosome 6 network, showing DEGs involved in each pathway. Color of outer bars on genes indicates log fold change. **c** Evaluation of ROS production in CV (blue) and DS-CV (red) by flow cytometry analysis. Data show the mean fluorescent intensity (MFI) of CV and DS-CV compared to positive control (CV + H_2_O_2_) (black). Data are represented as the mean ± standard deviations of at least three independent experiments. * = *p* < 0.05, ** = *p* < 0.01. **d** The expression of MSC-markers after the exposure of pro-inflammatory cytokines. The expression level of each marker was determined by qPCR and compared in CV (blu) and in DS-CV (red) exposed for 48 h to TNF-α and IFN-γ. **e** Quantitative polymerase chain reaction for the expression of genes associated with MSC-immunosuppressive potential. The *Pge2, Cox2, Il-6, Tgf-β and Tnf-α* genes expression were compared between CV (blue) and DS-CV (red) after 24 and 48 h of exposure to *TNF-α* and *IFN-γ*. The qPCR data are represented as fold change compared with the expression levels found in control cells (CV, baseline). *Significant and **highly significant differences compared with CTRL at *p* < 0.05 and *p* < 0.01, respectively

Figure 4a shows the largest network of DEGs located on chromosome 21 and chromosome 6 containing a total of 60 nodes and 110 edges, with 20 nodes up-regulated and 1 down-regulated on chromosome 21 and 20 up-regulated and 6 down-regulated on chromosome 6. Immune-related genes located on chromosomes 6 and 21 showed the most significant transcription dysregulations (Fig. 4b). Specifically, *Ifnar1* (FC = 0.815, P adj = 0.005) and *Ifnar2* (FC = 1.013, Padj = 0.004) (chromosome 21) were both up-regulated. An up-regulation of all components of the MHC (class I for *Hla-b* and class II for the other four), including *Hla-b* (FC = 2.836, Padj = 1.24e−6) and *Hla-dma* (FC = 1.980, Padj = 0.012) and down-regulation of *Hla-dra* (FC = -4.757, Padj = 0.028), *Hla-dpa1* (FC = -6.012, Padj = 0.0104) and *Hla-dpb1* (FC = -6.278, Padj = 0.0029) were all from chromosome 6. Significantly enriched genes and processes corresponding to chromosomes 21/6 network are also shown. The enrichment of genes located in the MHC protein complex (FDR = 3.11e−6) was further confirmed by enrichment analysis. The innate immune response (FDR = 3.4e−2) and cellular response to cytokine stimulus (FDR = 7.7e-3) pathways were also enriched, indicating that immune response function is altered in DS-CV cells compared with CV cells.

To confirm the dysregulation for the inflammatory genes found in the NGS data, we analyzed the cellular environment through the measure of intracellular ROS levels and expression of inflammatory genes. The measurement of intracellular ROS levels production serves as cell signaling molecules for normal biologic processes. DS-CV displayed a prompt and marked ROS production compared to their CV counterparts (Fig. 4c). Specifically, DS-CV showed significantly increased levels of ROS (670.4 ± 52.1 *p* < 0.01) compared not only to CV (502.5 ± 49.3) but also to CV treated with H_2_O_2_ (565 ± 17 *p* < 0.5). The exposure to an inflammatory environment, induced by the presence of tumor necrosis factor-α and interferon-γ (TNF-α and IFN-γ), resulted in a statistically significant increase in the expression of MSC-associated markers compared to their baseline levels. These changes were remarkable in DS-CV. Specifically, compared to CV after 48 h of treatment, the expression levels found in DS-CV were as follows: 1.73 ± 0.05-fold (*p* < 0.05) for *Cd105*, 3.61 ± 0.32-fold (*p* < 0.05) for *Cd90*, 5.07 ± 0.18-fold (*p* < 0.01) for *Cd73*, 5.85 ± 0.37-fold (*p* < 0.01) for *Cd44* and 8.90 ± 0.18-fold (*p* < 0.01) for *Cd29*, respectively (Fig. 4d). MSCs from DS-CV were also more prone than CV to react to pro-inflammatory cytokines (TNF-α and IFN-γ) as demonstrated by the evaluation of the immunosuppressive genes tested. Twenty-four hours after the treatment started, the levels of mRNA of *Pge2 (Prostaglandin E2)* and *Tgf-β (Transforming growth factor beta 1)* were up-regulated when compared to CV: 3.21 ± 0.25-fold (*p* < 0.05) and 2.92 ± 0.009-fold (*p* < 0.05), respectively (Fig. 4e). Moreover, after 48 h of treatment, the expression of immunosuppressive genes was increased progressively from a median fold of 3.83 (± 0.31), 2.38(± 0.28), 3.21(± 0.31) increase for *Pge2, Cox2 (cyclooxygenase 2), Tgf-β* (*p* < 0.05), respectively. Expression levels of the pro-inflammatory molecule *Tnf-α* were also found up-regulated in DS-CV compared to CV with values assessed around 14 ± 0.5-fold (*p* < 0.01) and 103(± 0.06)-fold increase at 24 and 48 h, respectively.

### Inflammatory genes increase on monocytic cells after DS-CV conditioned media

**Fig. 5:**
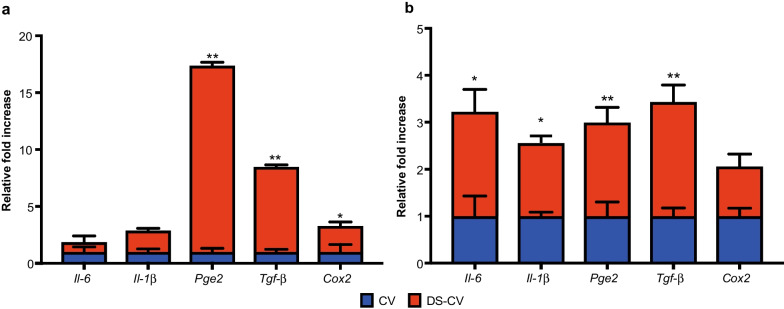
Inflammatory genes increase on monocytic cells after DS-CV conditioned media (CM). qPCR was used to observe the expression genes in monocytic cells (THP-1 cell line) following 24 (**a**) and 48 (**b**) hours of exposure to the media conditioned by CV and DS-CV (CM CV and CM DS-CV, respectively). Data are represented as fold change compared with the expression levels found in control cells (CV, baseline). *Significant and **highly significant differences compared with CTRL at *p* < 0.05 and *p* < 0.01, respectively

To confirm the immunosuppressive capability of CV and DS-CV and understand whether this process could be mediated by the paracrine signals they release, media conditioned by the presence of CV and DS-CV (CM CV and CM DS-CV, respectively) was added to the THP1 monocytes. The effect of CM CV and CM DS-CV was assessed by analyzing the expression of anti- and pro-inflammatory genes by THP-1 at 24 and 48 h. As can be observed in Fig. 5a, after 24 h of exposure to CM DS-CV, a statistically significant increase in the expression of anti- *(Pge2, Tgf-α* and *Cox2)* inflammatory genes was observed in THP-1 compared to the treatment with CM CV. A 16 ± 0.3-fold increase was observed for *Pge2* (*p* < 0.01), a 7.4 ± 0.1-fold increase (*p* < 0.01) was found for *Tgf-β* and 2.2 ± 0.3-fold increase (*p* < 0.05) was assessed for *Cox2*. No statistical differences between the two groups were observed in the expression of pro-inflammatory genes, Interleukin -6 (*Il-6)* and Interleukin-1β (*Il-1β)*. These values were found to drastically drop after 48 h of exposure in CM DS-CV although significant differences were still maintained compared to their CM CV counterparts for the expression of *Il-1β* (1.62 ± 0.1-fold *p* < 0.05), *Pge2* (1.99 ± 0.4-fold *p* < 0.01) and *Tgf-β* (2.4 ± 0.3-fold *p* < 0.01) (Fig. 5b). On the other hand, an increase in the expression levels of *Il-6* (2.22 ± 0.47-fold, *p* < 0.05) was noted in THP-1 treated with CM DS-CV, compared to those exposed to CM CV.

## Discussion

In this study, we proposed that MSCs derived from a gestational source, the chorionic villi, isolated from fetuses diagnosed with DS, could be used as a means of performing deep, functional genomic-informed studies of the underlying clinical complexity of Down syndrome phenotypes. In particular, the availability of CV samples during prenatal diagnosis allows for the identification of patient-specific genetic alterations and opens the opportunity to develop therapeutic approaches that might optimize health outcomes in these individuals [12].

NGS analysis unraveled molecular differences between CV-DS and their euploid counterparts that may contribute to the pathogenesis of DS and influence the patient’s susceptibility to one of the many associated disorders (e.g., heart diseases, leukemia and neurological disorder such as epilepsy, and Alzheimer’s disease) that compromise the quality of life of DS patients [33]. These differences include the participation and the correlation of several chromosomes in addition to the chromosome 21, which play a role in the genetic imbalance associated with this condition. While early transcriptomic studies performed on primary culture of amniocytes [6, 36] have already revealed significant transcriptional alterations in the regions of other chromosomes, including 1, 6, 11, 16, 17 and 19 [16], the present study proposes additional chromosomes as responsible for the specific signatures associated with DS, namely chromosome 2 and 22, and highlights the role of new DEGs located on chromosome 6 as responsible for proliferation, inflammation and immune response in addition to chromosome 21 which to characterize to develop of additional comorbidities in Down syndrome. Our data showed the presence of DEGs in different chromosomes, such as the chromosome 2, 6 and 22, in line with observations from Letourneau et al. who suggested that the global gene expression in DS is based on differences in chromatin topology generating gene expression dysregulation domains (GEDDs) [37]. Other groups consider DEG heterogeneity as a stochastic mechanism occurring among individuals and within the same individual, which tightly depends on the cell type and tissue analyzed [12, 38]. Several factors contribute to such heterogeneity, including the trisomy-induced changes which alter cellular function and its interaction with surrounding cells, ultimately leading to a secondary distortion of function or gene expression in neighboring cells, and epigenetic factors [12]. The latter have been proposed to be involved in correct chromosome organization and loss of chromatin-accessibility that ultimately play a role in the neurodevelopmental pathogenesis of DS [39]. All these factors highlight the differences in DEGs obtained in different studies and they could also emphasize the important role of other chromosomes in the DS-associated genetic imbalance, besides the well-known extra copy of chromosome 21 and the recently suggested chromosome 6 [37].

Our findings show that most DEGs identified in CV-DS compared to CV are mainly involved in the regulation of the innate immune response and cell response to oxidative stress, which are located on chromosomes 6 and 21. In particular, NGS analysis showed an up-regulation of interferon receptors (IFNRs)-associated genes (*Ifnr1* and *Infr2*) in chromosome 21 and *Ifngr1* in chromosome 6 [37]. To date, IFN ligands are produced by cells in response to a variety of insults (such as IFN-α, β or γ) activating the signaling pathway for the expression of pro-inflammatory cytokines (e.g., IL-1β, TNF-α and IL-6) [38]. In vitro experiments confirmed these observations, as demonstrated by the marked increase of the levels of inflammatory genes in CV-DS compared to CV following the treatment with pro-inflammatory cytokines. This aspect is of a particular interest, as hyperactive IFN signaling has been shown to have profound negative impacts on human development, making it vulnerable at mostly viral or bacterial infections and thus determining DS vulnerability [11]. In addition, a study conducted by Sullivan et al. revealed a condition of chronic autoinflammation in DS with elevated levels of important inflammatory cytokines linked to IFN signaling, such as IL-6, TNF-α and monocyte chemoattractant protein-1 (MCP-1) [38], which might explain why DS individuals are more prone to develop additional comorbidities [40].

Further investigations have shed light on the involvement of transcriptional variations of *App*, *Ifnr1* and *Infr2* genes in brain neuropathologies and mental retardation in DS [41]. RNA-Seq performed by us revealed the up-regulation of another set of genes mapped on chromosome 6 that are involved in mitochondrial dysfunctions [42], the superoxide dismutase 2 genes (*Sod2*). It is known that Cu/ZnSOD activity is increased in DS patients, with the consequent production of more hydrogen peroxide than catalase and glutathione peroxidase can catabolize. This, in turn, gives rise to an oxidative stress positive feedback that induces mitochondrial dysfunction and impairs the respiratory complex enzymes, ultimately leading to an increased ROS production [43–45]. As reported in the literature, pathways involved with an excessive production of ROS by cells during aerobic respiration play important roles in the pathogenesis of a plethora of conditions, including cardiovascular diseases, hypertension and atherosclerosis [42]. The alteration of cellular redox status and the increase of the ROS generation has been associated to the up-regulation of β-amyloid precursor protein (APP) and to enhance DS individual’s vulnerability to various stress factors [46]. The *App* gene is located on chromosome 21 and its protein is expressed in glial cells and thus, in neuronal axons. APP protein is known to be localized in mitochondria and it is involved in regulation of secretory pathways. The overexpression of APP in neuronal cells is a common factor in DS patients and it is related to extra copy of chromosome 21 [47]. According to literature, we found an up-regulation in the APP-encoding gene present on chromosome 21. The disruption of normal signaling function of APP is reported to cause cell cycle abnormalities in neurons and to be associated with neurodegeneration and consequent dementia in DS. Indeed, approximately 50 to 70% of individuals with DS develop dementia by age 60 to 70 years [46]. Different studies report the neurological disease [46] as Alzheimer [48] is due to early amyloid plaques formation as consequent overexpression related to DS clinical conditions.

DEGs located on chromosome 6 also include *Tnf* and *Hla*, besides *Sod2* and *Ifngr1*. The up-regulation of these genes is correlated with the induction of signal transducer and activator of transcription (STATs) and mitogen-activated protein kinase (MAPKs) pathways [49], which are associated with inflammatory conditions. The overexpression of inflammatory genes creates the chronic inflammation status within cells [50]. Interestingly, our transcriptomic profile showed a down-regulation of genes associated with MHC-II class genes and the Kinesin family member C1 (*Kifc1*) which are again located on chromosome 6. We found three *Hla*-genes (*Hla-dra, Hla-dpa1* and *Hla-dpb1*) related with MHC which are involved in the immune suppression [49–51] and play a crucial role in tumor immune escape [52]. A low expression of HLA-class II molecules, as in our study with the DS sample, has been correlated with a higher risk of metastasis and associated to several tumors, such as sarcomas, hepatocellular carcinomas [53], B cell lymphomas, leukemia and autoimmune disorders [54]. Furthermore, the decreased susceptibility of the HLA-class II molecules has been linked to predisposition to develop type 1 diabetes a common pathology in DS [55]. Through GSEA analysis we found an up-regulation of several genes coding the histone cluster which make the nucleosome structure (*Hist1-h1c, Hist1-h2ac, Hist1-h2bo, Hist1-h2bn, Hist1-h2bc* and *Hist1-h4h*) located on chromosome 6. According to the literature, a correct nucleosome structure formation is required for the accurate assembly of chromatin as well as for the proper cell division and growth. Their overexpression has been shown to block transcription in vitro by triggering chromatin aggregation and to increase the incidence of mitotic chromosome loss, DNA damage, cell growth inhibition, inflammatory status promotion and cell toxicity [56]. The overexpression of histone gene *Hist1-h1c* along with the *Sirt1* gene has also been correlated to the development of diabetes, which is a common condition secondary to DS [57]. As mentioned above, the overexpression of inflammatory genes can define a framework of increased production of cytokines and signaling activity for the immune system and increase the risk for DS individuals to develop an autoimmune disease [58]. Concomitantly, the GSEA analysis (Go process, KEGG and Reactome) showed the presence of DEGs associated with the cell cycle, on chromosomes 2, 6 and 22. Interestingly, an altered expression of centromere protein (*Cenp*) genes and cell cycle checkpoints (i.e., *Cenpm, Mcm5, Gtse1 and Cdc45*) was found. CENPs are proteins expressed in the formation of centromeric structure and key elements for the formation of a correct kinetochore, essential for mitosis and whose dysregulation induces the mitosis alteration causing the alteration of the whole cell cycle [59, 60].

Two more DEGs were identified in DS-CV, *Bub1* and *Cdk1*. During mitosis, *Bub1* is required to regulate chromosome segregation and maintain telomeric genome integrity [61]. It also plays an important role in the TGF-β signaling [62]. *Cdk1* gene is involved in cell cycle progression and mitochondrial metabolism [63]. Our functional studies have confirmed these observations, showing a significant reduction of the proliferative potential and an impairment in cell cycle in DS-CV samples compared to their euploid counterparts. Experimental data are further corroborated by other gene sets we identified on chromosome 22. They include the *Cdc45* gene, a component of the CMG (CDC45-MCMs-GINS) complex, whose expression is related to cycle progression. In case of loss of the expression levels, *Cdc45* determines the inhibition of DNA replication and G1-phase arrest with consequently inhibition of the cell proliferation [64]. In addition to the molecular justification provided above, impairment in cell cycle could also be associated with the increasing expression of the CD73 we found in DS-CV. To date, CD73 is an ectoenzyme involved in several cell activities, including tissue homeostasis and support of epithelial cell transport [65]. *Cd73* and *Cenp* genes could interfere with the normal cell cycle, confirming the alteration in the cell cycle observed in DS-CV [66]. Taken together, our data reveal potential gene patterns which could influence the occurrence of diseases related to DS. The pathways we found dysregulated in this study are involved in cell cycle and in proliferation activity, which have been linked with neuropathologies, including Alzheimer's disease, which represents one of the typical DS comorbidities [67]. Furthermore, the expression of CD73 strongly correlates with the responses to immunity and cancer, since this protein is involved in the reinforcement of lymphocyte–endothelium interactions, inhibition of macrophage and mesenchymal cell-mediated inflammation [68]. CD73 induction is associated with the increase of the expression of pro-inflammatory cytokines (i.e., IL-6 and IL-27), high levels of oxidative stress and hypoxia, among others [69]. With several molecular moieties being involved in the greater reactivity of DS-CV, we hypothesized that the DS-CV phenotype could be reflected in their capability to release bioactive factors acting as mediators of cell-to-cell communication. To test this hypothesis, we investigated the immune-suppressive and immune-modulatory properties of DS-CV in comparison with CV by exposing monocytic cells (THP-1 cells) to media conditioned by each cell type. Gene expression performed on THP-1 cells grown in the presence of DS-CV CM, showed a significant increase in the levels of pro (*Il-6* and *Il*1β)- and anti (*Pge2 Tgf-*β and *Cox2*)-inflammatory genes at 24 and 48 h, suggesting a potentially different molecular composition in the paracrine factors they release [28]. Among them, extracellular vesicles (EVs) play a crucial role in cellular signaling as they are taken up by recipient cells, determining the activation or inhibition of several biological processes including cell proliferation, differentiation, apoptosis and the immune response [70]. MSC-derived EVs are extremely relevant as diagnostic tools and for the development of therapeutic cell-free drugs [71, 72]. The reaction to external stimuli leads to a feedback response via cellular or exosomal signaling consequently to damages or altered pathways. Gauthier et al. detected an enhanced endosomal release into extracellular space via exosomes as a protective effect under pathological conditions [73]. According to this study, our GSEA analysis found DEGs on chromosome 2 and 6 that are involved in the regulation of endoplasmic reticulum (ER) with a key role in protein folding. The inflammatory state observed during Down syndrome state could therefore influence the ER creating an imbalance between the protein-folding load and the capacity of the ER, causing unfolded or misfolded proteins [74]. Based on the tight correlation between the inflammatory response and the ER stress, we observed in DS-CV an excess of metabolic factors being released such as lipids, glucose and cytokines and neurotransmitters. In addition, ER controls cellular metabolism by regulating protein synthesis and secretion, as well as triglyceride and cholesterol biosynthesis. The ER stress could then trigger an excessive lipid accumulation as well as an increase of glucose levels associated with both obesity and diabetes [75]. Furthermore, protein aggregation resulted from ER stress has been associated with the Alzheimer and Parkinson’s disease and confirmed by the GSEA analysis we performed, identifying the *App* gene among those involved in the endoplasmic reticulum which is reported to play a role in the development of the Alzheimer’s disease, another typical secondary condition in DS [76].

## Conclusions

This study provides a deeper investigation on the role of chromosomes 21, 6, 2 and 22 play in determining the complex pathological phenotype of DS individuals. The transcriptomic analysis performed on MSCs isolated from CV obtained for prenatal diagnosis of genetic disorders allowed us to expand our knowledge of this complex clinical condition. Our data demonstrate that while trisomy 21 does not interfere with standard stem cell features, it does promote an imbalance in the expression of genes that are associated with essential cellular processes, impairing cell cycle at a structural and functional standpoint, and dysregulating the immune response under specific stimuli, ultimately leading to chronic disorders commonly associated with DS. The newly identified DEGs and their locations represent potential new targets for the development of therapeutic strategies against DS morbidities. Furthermore, the insights obtained from the in vitro evaluation of the paracrine signals released by DS-CV compared to CV provide a solid basis for their use as diagnostic biomarkers as their production and content is expected to reflect divergences across genetically induced pathological conditions. While datasets generated here are promising they call for a subsequent investigation based on a larger number of patient-derived samples to provide stronger evidence and draw a definitive conclusion.

### Supplementary Information

**Additional file 1. **Quantitative PCR between CV and DS-CV performed to evaluate the expression gene levels of collagen I at 7 and 14 days of osteogenic induction. Results show an increased expression in both CV and DS-CV. Data are showed as the mean (±SD) of three biological replicates.

**Additional file 2: Figure S1.** Heatmaps of Euclidean distance between CV and DS-CV. **a** Heatmap of Euclidean distance scores of normalized genes counts on chromosome 2 for DS-CV versus CV. Dendrograms show hierarchical clustering results. **b** Heatmap of Euclidean distance scores of normalized genes counts on chromosome 6 for DS-CV versus CV. **c** Heatmap of Euclidean distance scores of normalized genes counts on chromosome 22 for DS-CV versus CV. Dendrograms show hierarchical clustering results.

**Additional file 3: Table S1.** The list of differential expressed genes (DEGs) in CV and DS-CV samples.

**Additional file 4: Figure S2.** Gene Set Enrichment Analysis (GSEA) for all genes (GO process, KEGG pathway and Reactome pathway). GSEA highlighted ranks genes by log fold change to identify up- and downregulated pathways and processes. In GO process (**a**), KEGG (**b**) and Reactome pathway (**c**) cell cycle processes or cell cycle pathways related are significantly down-regulated.

**Additional file 5: Table S2.** Gene set enrichment analysis (GSEA) for CHr21_2_ (GO process, KEGG pathway, Reactome pathway).

**Additional file 6: Table S3.** Gene set enrichment analysis (GSEA) for CHr21_6_ (GO process, KEGG pathway, Reactome pathway).

**Additional file 7: Table S4.** Gene set enrichment analysis (GSEA) for CHr21_22_ (GO process, KEGG pathway, Reactome pathway).

**Additional file 8: Figure S3.** Chromosome cytoband enrichment. The GSEA applied to all differentially expressed genes identify the enriched genomic locations (chromosome cytobands) from the msigdb hallmarks collections. **a** Three cytobands outside of chromosome 21 were found to be enriched with FDR < 0.1. The band chr22q11.25 was up-regulated and the bands, chr2q31.1 and chr6p21.32 were down-regulated. **b** Part of the detected DEGs mainly linked to chromosomes 2, 22 and 6.

**Additional file 9: Figure S4.** CV and DS-CV differentiative potential. Representative images showing CV and DS-CV undergoing osteogenic (**a**) and adipogenic (**b**) differentiation after 7 and 14 days of induction. Osteogenesis was confirmed by von Kossa staining to highlight mineral deposition and adipogenesis by Oil-red-O positive cytoplasmic neutral lipids. Expression levels of osteogenic markers, (the transcription factor RUNX-2 and osteocalcin, *Bglap*) and (the nuclear receptor *PPAR-g* and adiponectin, *Adipq*) were determined by quantitative RT-PCR after 14 days of induction. Data were normalized to the reference gene (*Gapdh*) and represented as fold change compared with the expression untreated CV or DS-CV. Values are mean ± SD (*n* = 3). Asterisks depict highly significant (*p < 0.01 and **p < 0.001).

## Data Availability

Raw and processed datasets have been deposited in NCBI’s Gene Expression Omnibus (GEO) (https://www.ncbi.nlm.nih.gov/geo) with accession reference GSE184450. Reviewer token: gtmjicaaxfetzup).

## Abbreviations

A/A: Antimycotic–antibiotics
ADIPQ: Adiponectin
APC: Allophycocyanin
APP: β-Amyloid precursor protein
BGLAP: Bone gamma-carboxyglutamate protein
BSA: Bovine serum albumin
CENP: Centromere protein
CFU-F: Colony forming unit—fibroblastic like
COX2: Cyclooxygenase 2
CV: Chorionic villi
DEGs: Differentially expressed genes
DS-CV: Down syndrome chorionic villi
DS: Down syndrome
ER: Endoplasmic reticulum
EV: Extracellular vesicles
FBS: Fetal bovine serine
FDR: False discovery rates
FITC: Fluorescein isothiocyanate
GEDD: Gene expression dysregulation domains
GEO: Gene expression omnibus
GSEA: Gene set enrichment analysis
H2-DCFDA: 2′,7′-Dichlorodihydrofluorecein
HBSS: Hank’s balanced salt solution
HG-DMEM: High-glucose Dulbecco’s modified eagle medium
IL-1β: Interleukin 1 beta
IL-6: Interleukin 6
INF: Interferon gamma
iPSC: Induced pluripotent stem cell
KIFC1: Kinesin family member C1
MAPK: Mitogen-activated protein kinase
MCP-1: Monocyte chemoattractant protein-1
MHC-II: Major histocompatibility complex
MSC: Mesenchymal stem cell
NCBI: National Center for Biotechnology Information
NGS: Next-generation sequencing
Padj: Adjusted *p*-value
PBS: Phosphate-buffered saline
PE: Phycoerythrin
PFA: Paraformaldehyde
PGE2: *Prostaglandin E2*
PI: Propidium iodide
PPAR-γ: Peroxisome proliferator-activated receptor gamma
RIN: Integrity number
ROS: Reactive of reactive oxygen species
RPMI: Roswell Park Memorial Institute-1640
RUNX-2: RUNX family transcription factor 2
SD: Standard deviation
SOD2: Superoxide dismutase 2
STAT: Signal transducer and activator of transcription
TFG-β: Transforming growth factor beta 1
THP-1: Acute monocytic leukemia
TNF-α: Tumor necrosis factor-alpha
