# Supplementary material for: Transcriptomic analysis of stem cells from chorionic villi uncovers the impact of chromosomes 2, 6 and 22 in the clinical manifestations of Down syndrome
Source: Stem Cell Res Ther. 2023 Sep 23;14:265. doi: 10.1186/s13287-023-03503-4 (PMC10517537; doi:10.1186/s13287-023-03503-4)
Supplement: Supplementary file 1 — Additional file 1. Quantitative PCR between CV and DS-CV performed to evaluate the expression gene levels of collagen I at 7 and 14 days of osteogenic induction. Results show an increased expression in both CV and DS-CV. Data are showed as the mean (±SD) of three biological replicates. [file 13287_2023_3503_MOESM1_ESM.docx]

**Supplementary Material #2**

**Reference number:** CRT-D-23-00309R2
**Manuscript title:** Transcriptomic analysis of stem cells from chorionic villi uncovers the impact of chromosomes 2, 6, and 22 in the clinical manifestations of Down Syndrome.
**Authors:** Salvatore Vaiasicca; Gianmarco Melone; David W. James; Marcos Quintela; Alessandra Preziuso; Richard H. Finnell; Robert Steven Conlan; Lewis W Francis; Bruna Corradetti

Quantitative PCR performed to evaluate the expression levels of collagen I on the remaining cDNA samples at 7 and 14 days of osteogenic induction. Results show an increased expression in collagen type I in both CV and DS-CV upon exposure to inducing media. Data are normalized to expression values obtained from untreated counterparts and shown as the mean (±SD) of three biological replicates.
