# Supplementary figures and images for: Transcriptomic analysis of stem cells from chorionic villi uncovers the impact of chromosomes 2, 6 and 22 in the clinical manifestations of Down syndrome
Source: Stem Cell Res Ther. 2023 Sep 23;14:265. doi: 10.1186/s13287-023-03503-4 (PMC10517537; doi:10.1186/s13287-023-03503-4)

**a****Chromosome 2 genes**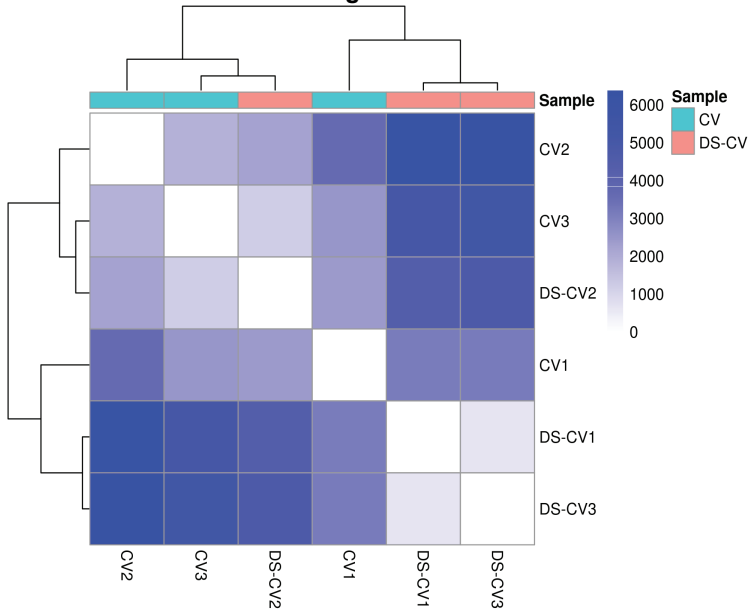**b****Chromosome 6 genes**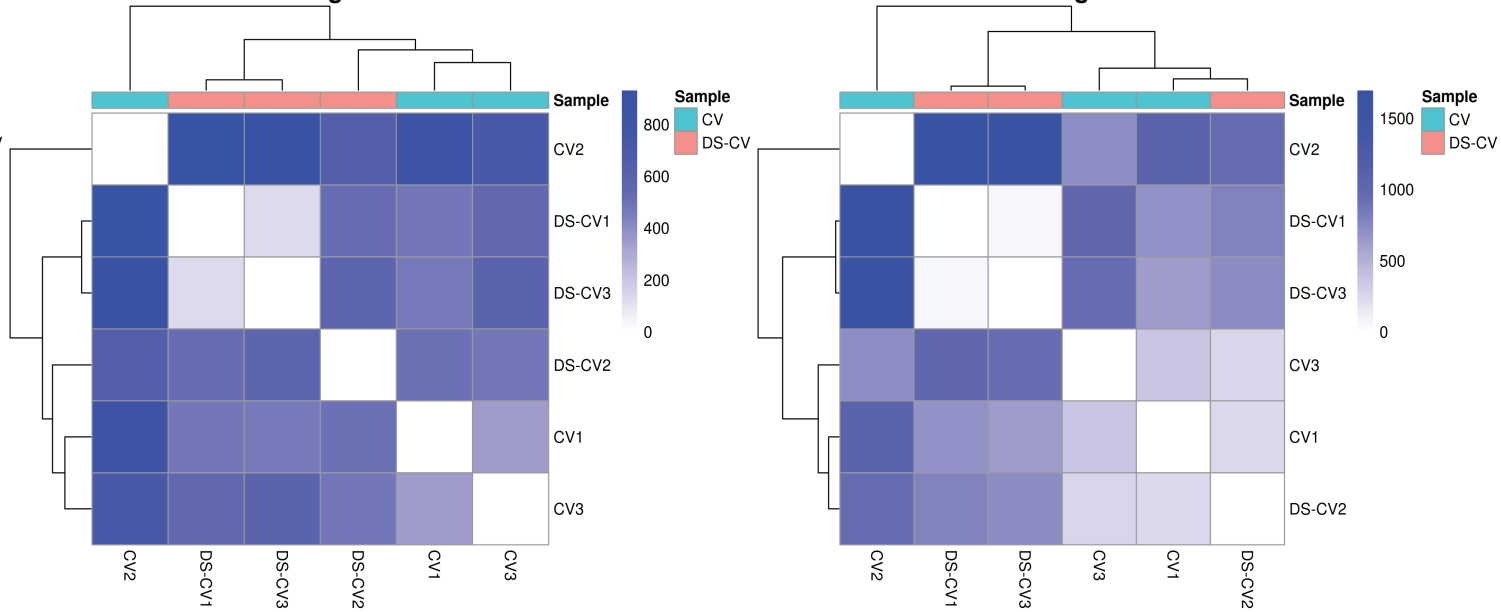**c****Chromosome 22 genes**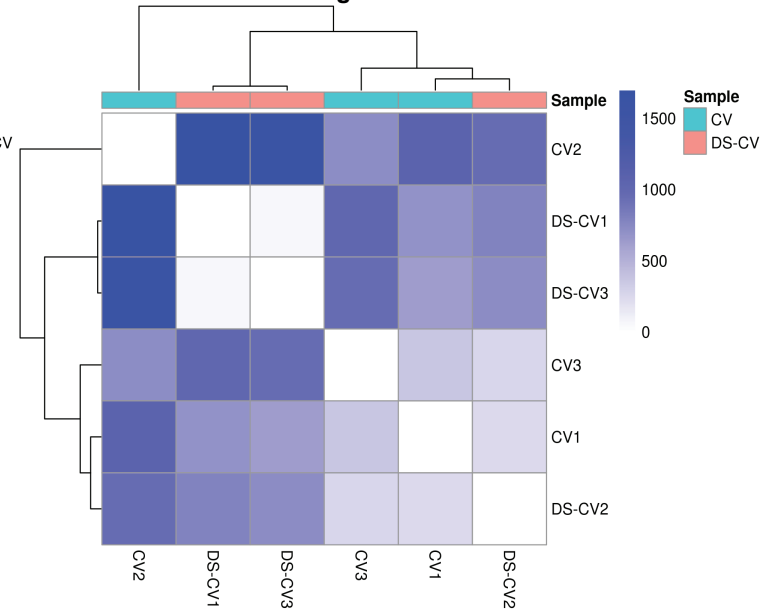

Supplement: Supplementary file 2 — Additional file 2: Figure S1. Heatmaps of Euclidean distance between CV and DS-CV. a Heatmap of Euclidean distance scores of normalized genes counts on chromosome 2 for DS-CV versus CV. Dendrograms show hierarchical clustering results. b Heatmap of Euclidean distance scores of normalized genes counts on chromosome 6 for DS-CV versus CV. c Heatmap of Euclidean distance scores of normalized genes counts on chromosome 22 for DS-CV versus CV. Dendrograms show hierarchical clustering results. [file 13287_2023_3503_MOESM2_ESM.pdf]

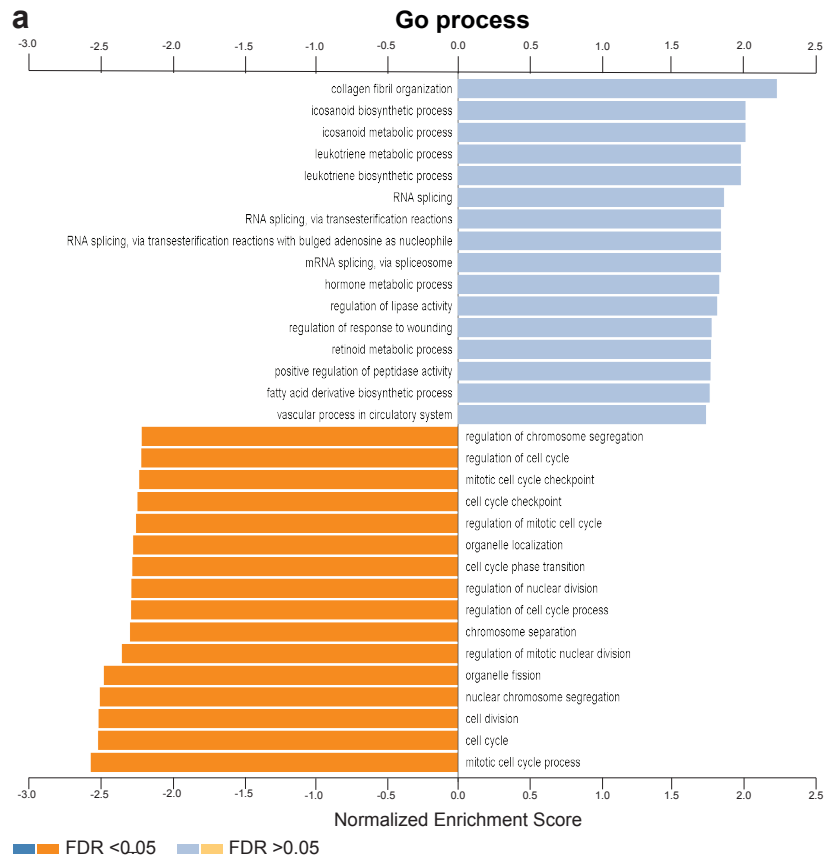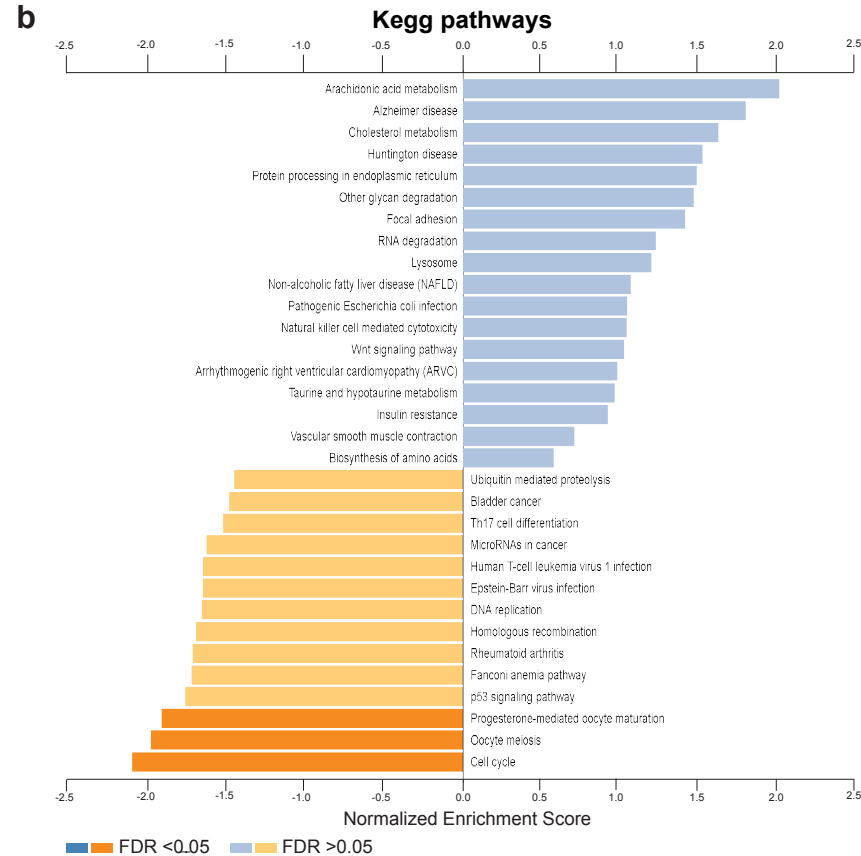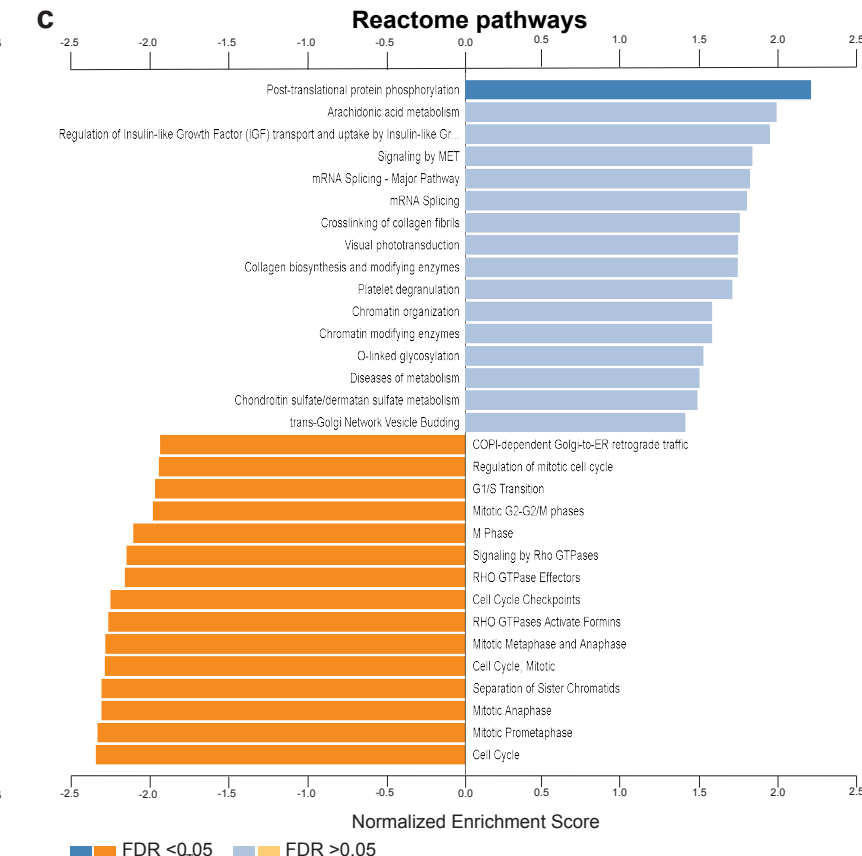

Supplement: Supplementary file 4 — Additional file 4: Figure S2. Gene Set Enrichment Analysis (GSEA) for all genes (GO process, KEGG pathway and Reactome pathway). GSEA highlighted ranks genes by log fold change to identify up- and downregulated pathways and processes. In GO process (a), KEGG (b) and Reactome pathway (c) cell cycle processes or cell cycle pathways related are significantly down-regulated. [file 13287_2023_3503_MOESM4_ESM.pdf]

**a****7 days****14 days**

CV

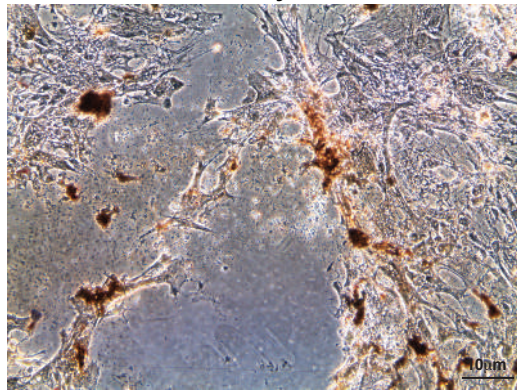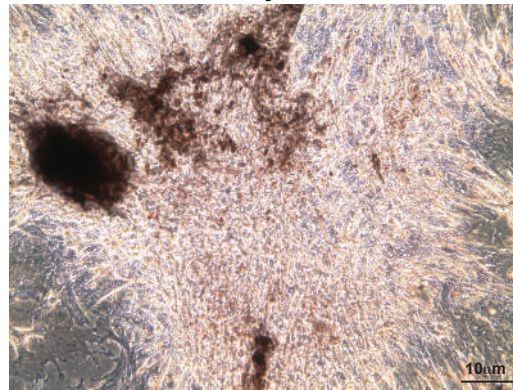

DS-CV

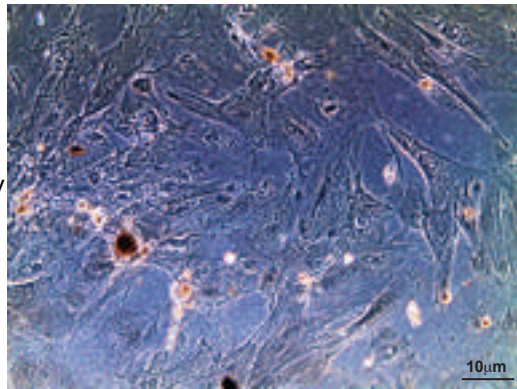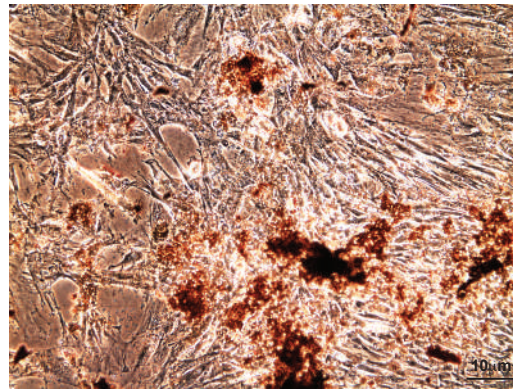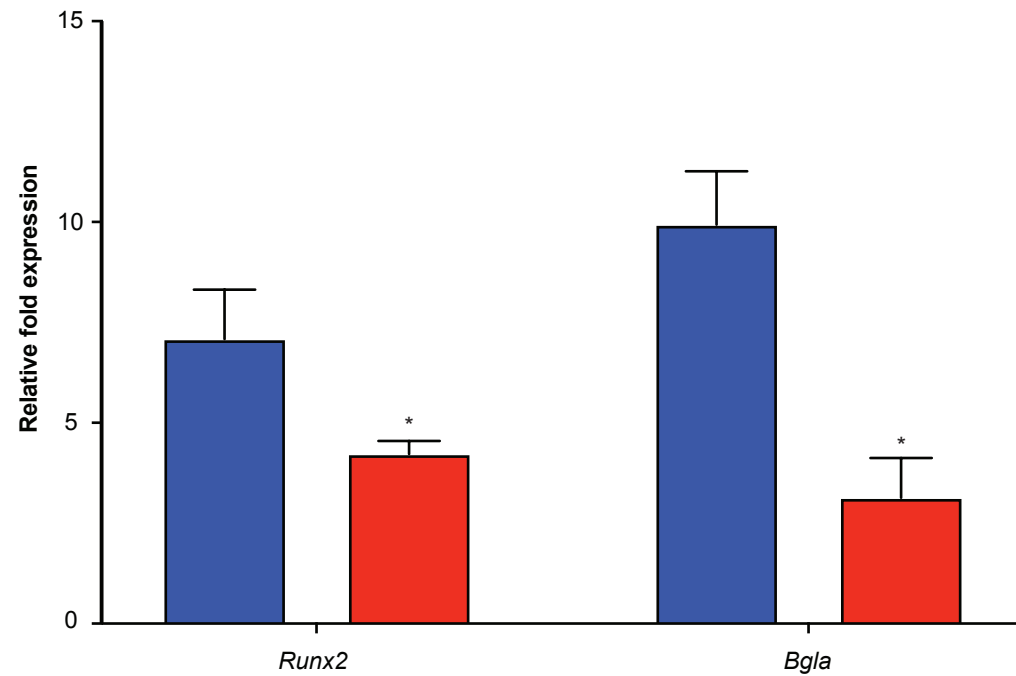**b****7 days****14 days**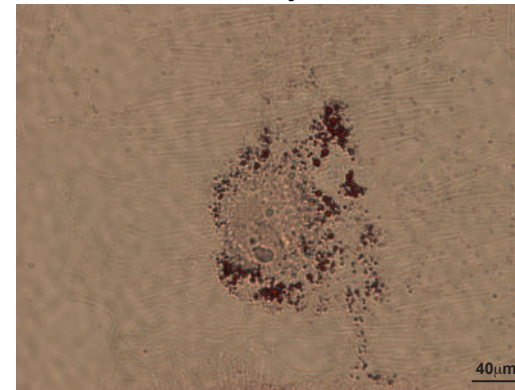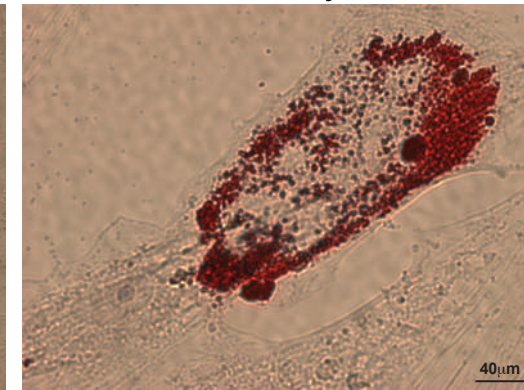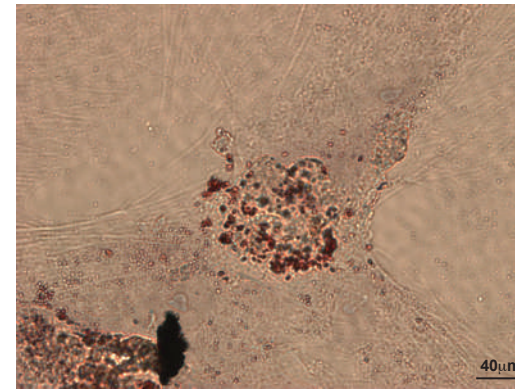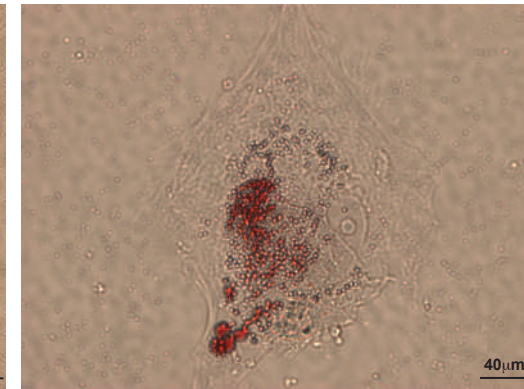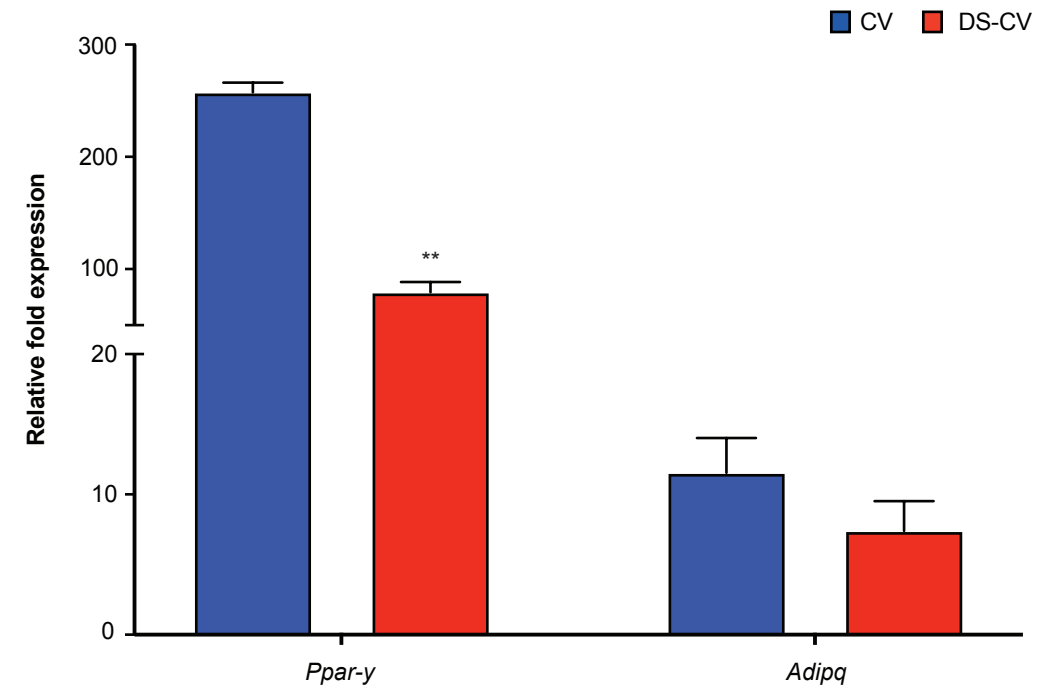

Supplement: Supplementary file 9 — Additional file 9: Figure S4. CV and DS-CV differentiative potential. Representative images showing CV and DS-CV undergoing osteogenic (a) and adipogenic (b) differentiation after 7 and 14 days of induction. Osteogenesis was confirmed by von Kossa staining to highlight mineral deposition and adipogenesis by Oil-red-O positive cytoplasmic neutral lipids. Expression levels of osteogenic markers, (the transcription factor RUNX-2 and osteocalcin, Bglap) and (the nuclear receptor PPAR-g and adiponectin, Adipq) were determined by quantitative RT-PCR after 14 days of induction. Data were normalized to the reference gene (Gapdh) and represented as fold change compared with the expression untreated CV or DS-CV. Values are mean ± SD (n = 3). Asterisks depict highly significant (*p < 0.01 and **p < 0.001). [file 13287_2023_3503_MOESM9_ESM.pdf]
