# Supplementary material for: Transcriptomic analysis of stem cells from chorionic villi uncovers the impact of chromosomes 2, 6 and 22 in the clinical manifestations of Down syndrome
Source: Stem Cell Res Ther. 2023 Sep 23;14:265. doi: 10.1186/s13287-023-03503-4 (PMC10517537; doi:10.1186/s13287-023-03503-4)
Supplement: Supplementary file 8 — Additional file 8: Figure S3. Chromosome cytoband enrichment. The GSEA applied to all differentially expressed genes identify the enriched genomic locations (chromosome cytobands) from the msigdb hallmarks collections. a Three cytobands outside of chromosome 21 were found to be enriched with FDR < 0.1. The band chr22q11.25 was up-regulated and the bands, chr2q31.1 and chr6p21.32 were down-regulated. b Part of the detected DEGs mainly linked to chromosomes 2, 22 and 6. [file 13287_2023_3503_MOESM8_ESM.pdf]

a

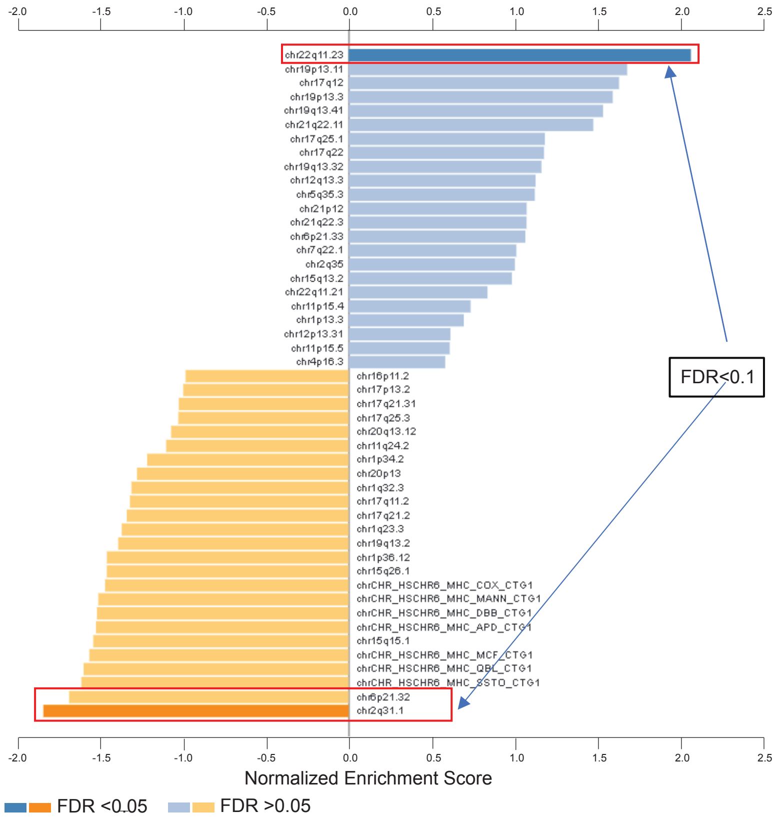

b

Genes chr22q11.25 (FDR 2.04e-2)

Gene Symbol	Gene Name	Score
SUSD2	sushi domain containing 2	5.3186
GGT5	gamma-glutamyltransferase 5	2.2187
GGT1	gamma-glutamyltransferase 1	2.1997
POM121L9P	POM121 transmembrane nucleoporin like 9, pseudogene	2.8368
BCRP3	breakpoint cluster region pseudogene 3	2.2673

Genes chr2q31.1 (FDR 1.50e-2)

Gene Symbol	Gene Name	Score
CDCA7	cell division cycle associated 7	-3.9253
SPC25	SPC25, NDC80 kinetochore complex component	-6.1592
HOXD8	homeobox D8	-5.4905
KLHL23	kelch like family member 23	-4.2275
PHOSPHO2-KLHL23	PHOSPHO2-KLHL23 readthrough	-4.2275
HOXD-AS2	HOXD cluster antisense RNA 2	-5.0771

Genes chr6p21.32 (FDR 9.54e-2)

Gene Symbol	Gene Name	Score
HLA-DRA	major histocompatibility complex, class II, DR alpha	-4.7566
HLA-DPB1	major histocompatibility complex, class II, DP beta 1	-6.2783
HLA-DPA1	major histocompatibility complex, class II, DP alpha 1	-6.0123
KIFC1	kinesin family member C1	-4.787
